# Ectopic parathyroid adenoma on sternocleidomastoid muscle: a case report

**DOI:** 10.3389/fonc.2024.1410057

**Published:** 2024-06-18

**Authors:** HuiMin Shan, ZhenPeng Jiang, Jin Xu, JingFa Li, XuSheng Zhu

**Affiliations:** ^1^ Department of Nuclear Medicine, Guangzhou First People’s Hospital, School of Medicine, South China University of Technology, Guangzhou, Guangdong, China; ^2^ Department of Pathology, Guangzhou First People’s Hospital, School of Medicine, South China University of Technology, Guangzhou, Guangdong, China

**Keywords:** primary hyperparathyroidism, ectopic parathyroid adenoma, Tc-99m-MIBI, SPECT, sternocleidomastoid muscle

## Abstract

A 54-year-old woman was admitted to the hospital with a left neck mass. Enhanced CT and ultrasound examinations revealed a lesion in the left sternocleidomastoid muscle. The patient undergone right thyroid lobe resection 8 years ago. Interestingly, the lesion on the sternocleidomastoid muscle, along with the left lobe of the patient’s thyroid, visually appears to form a displaced and complete thyroid in the early Tc-99m-MIBI parathyroid scintigraphy. Combined with Tc-99m-MIBI scintigraphy and abnormal PTH and blood calcium levels, the consideration was given to the lesion in the sternocleidomastoid muscle as an ectopic parathyroid adenoma. Subsequent surgical pathology confirmed this suspicion.

## 1 Introduction

Primary hyperparathyroidism (PHPT) is a common endocrine disorder characterized by hypercalcemia and elevated parathyroid hormone levels. Hyperparathyroidism affects 20 to 30 people per 100,000 people each year, and most cases are closely associated with parathyroid adenomas (1). Ectopic parathyroid adenomas (EPA) are rare causes of PHPT, comprising less than 5% of cases (2). They can be located anywhere from the base of the tongue to the mediastinum, commonly found in the superior mediastinum or posterior to the esophagus (3). Here, we report a rare case of an ectopic parathyroid adenoma due to its unusual location and distinctive imaging features.

## 2 Case report

**Figure 1:**
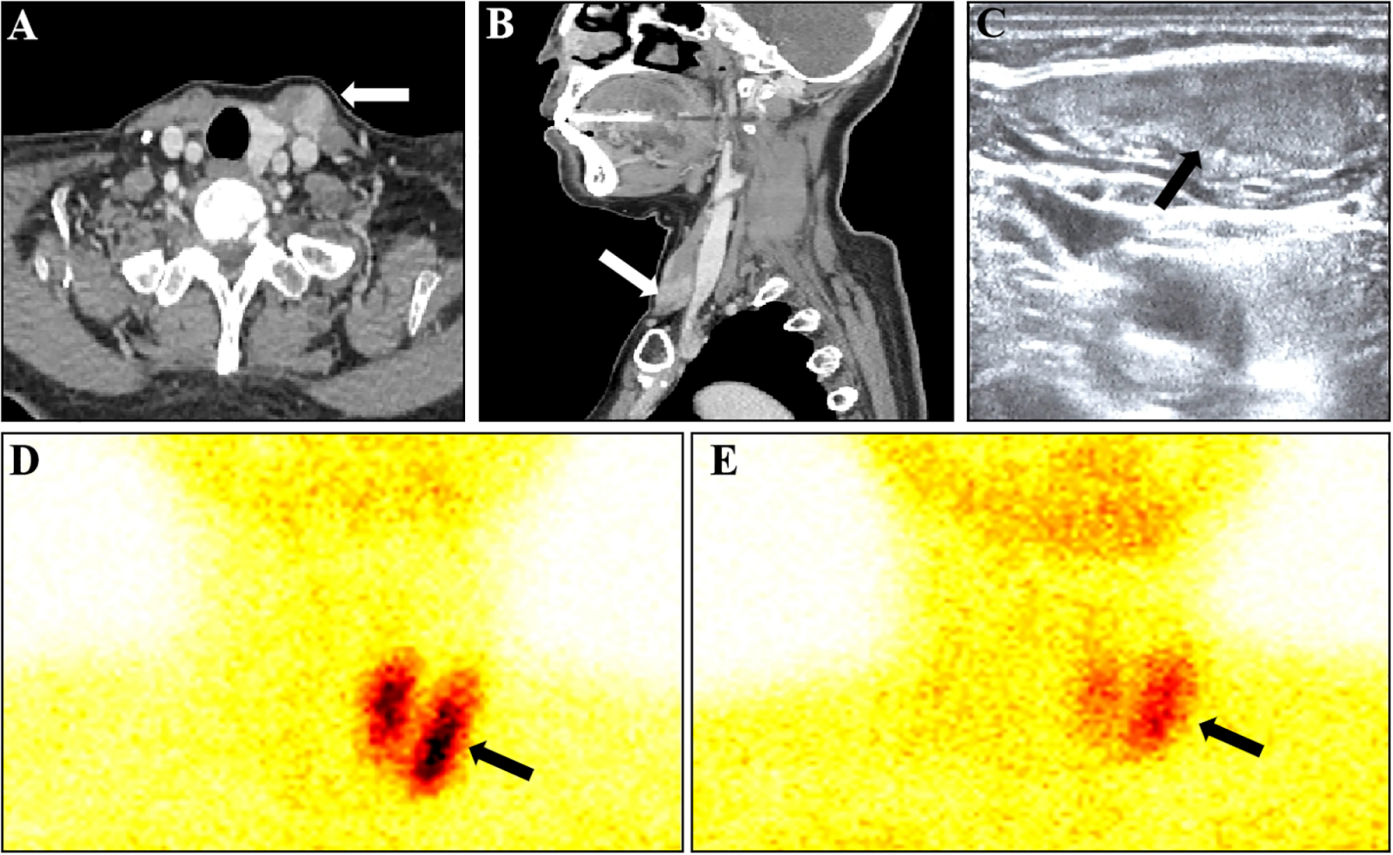
Various imaging findings of ectopic parathyroid adenoma in the sternocleidomastoid muscle. **(A)** Axial image in contrast-enhanced CT showed a soft tissue lesion (arrow). **(B)** Sagittal image in contrast-enhanced CT showed a soft tissue lesion (arrow). **(C)** Conventional ultrasound Showed a well-defined slightly hyperechoic mass. **(D)** Early (15 minutes) images in Tc-99m-MIBI scintigraphy showed a cord-like high uptake in the projection area of the left sternocleidomastoid muscle. **(E)** late (2 hours) images in Tc-99m-MIBI scintigraphy still showed a cord-like high uptake in the projection area of the left sternocleidomastoid muscle.

**Figure 2:**
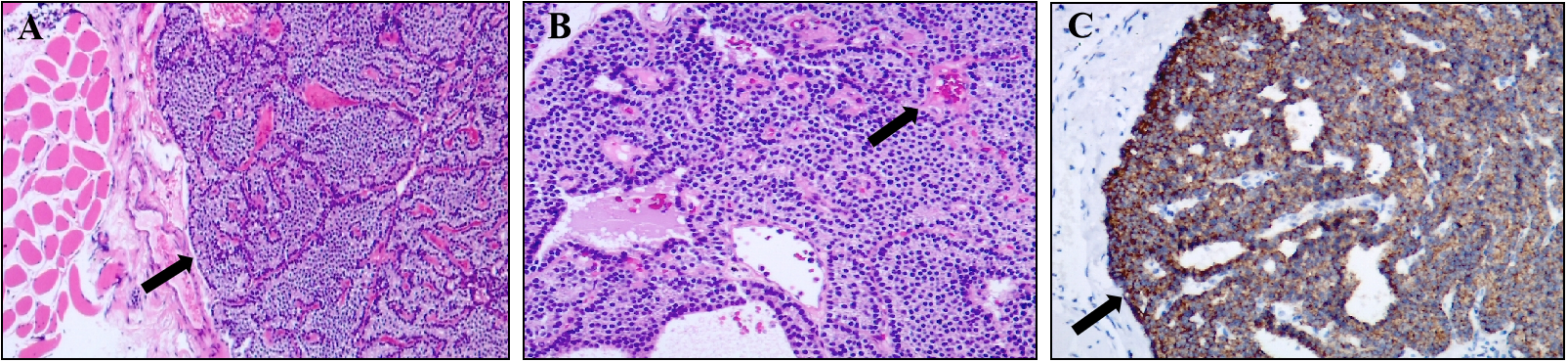
Pathological findings of ectopic parathyroid adenoma in the sternocleidomastoid muscle. **(A)** Image of hematoxylin-eosin (magnification, ×50) showed the mass occurred in striated muscle tissue. **(B)** Image of hematoxylin-eosin (magnification, ×100) showed conspicuous follicular structures, concomitantly with tumor cells exhibiting a palisade arrangement around blood vessels (arrow). **(C)** Immunohistochemistry result showed a strong positive expression of parathyroid hormone (magnification, ×100).

A 54-year-old female presented with a gradually enlarging left neck mass. Physical examination revealed a painless, firm, non-mobile 4 cm mass in the left neck. Axial (**Figure 1A**) and sagittal (**Figure 1B**) contrast enhanced CT images revealed a spindle-shaped soft tissue lesion with clear borders in the left sternocleidomastoid muscle, showing homogeneous hyperenhancement. Conventional ultrasound (**Figure 1C**) identified a well-defined slightly hyperechoic spindle-shaped lesion (measuring 40 mm × 17 mm × 9 mm) in the left sternocleidomastoid muscle with color Doppler displaying abundant blood flow signals, raising suspicion of a myogenic tumor. Subsequently, a Tc-99m-MIBI parathyroid scintigraphy was conducted. Early (**Figure 1D**, 15 minutes) and late (**Figure 1E**, 2 hours) images in Tc-99m-MIBI scintigraphy both exhibited well-defined, regularly shaped, cord-like high uptake in the projection area of the left sternocleidomastoid muscle. Interestingly, the lesion on the sternocleidomastoid muscle, along with the left lobe of the patient’s thyroid, visually appears to form a displaced and complete thyroid in the early Tc-99m scintigraphy. However, the patient had actually undergone right thyroid lobe resection 8 years ago due to nodular goiter. Blood tests revealed elevated serum calcium (2.92 mmol/L; reference range, 2.11–2.52 mmol/L) and parathyroid hormone (319 pg/mL; reference range, 15–65 pg/mL). And the serum creatinine levels (61 umol/L; reference range, 41–73 umol/L) were in the normal range. Urinary analyses revealed hypercalciuria, 15.44 mmol/24 hours (reference range, 2.5–7.5 mmol/24 hours). The patient has no history of osteoporosis or kidney stones. Based on imaging and laboratory examination results, there is a preoperative suspicion that the tumor of the sternocleidomastoid muscle originates from ectopic parathyroid tissue, with malignant potential not being excluded. Subsequently, the lesion measuring approximately 40mm× 20mm ×10 mm was successfully excised through surgery. Hematoxylin and eosin staining (**Figures 2A, B**) of the lesion showed the adenoma growing within the striated muscle, and conspicuous follicular structures, concomitantly with tumor cells exhibiting a palisade arrangement around blood vessels. Immunohistochemistry revealed a strong positive expression of parathyroid hormone (**Figure 2C**). Moreover, the expression of Ki-67 was less than 5%. Atypical features (4) such as cellular nests in a thickened connective tissue, trabecular growth, increased mitotic activity, atypical mitotic figures, coagulative necrosis, and a Ki-67 labeling index >5% were not identified in the pathology findings of our patient. Similarly, definitive criteria of malignancy (4) including angioinvasion, lymphatic invasion, perineural invasion, unequivocal invasion into adjacent structures, and histologically confirmed metastasis were absent. Therefore, the parathyroid adenoma ectopically located in the sternocleidomastoid muscle was confirmed by pathological findings. After surgery, the patient’s parathyroid hormone and blood calcium levels rapidly decreased to normal levels. Currently, the patient’s condition is good, with a favorable prognosis.

## 3 Discussion

PHPT is a disease characterized by the excessive secretion of parathyroid hormone, leading to hypercalcemia, osteoporosis, and urinary tract stones (5). Both orthotopic parathyroid adenomas and EPA can cause PHPT. Parathyroid adenomas located in ectopic sites may present with different clinical manifestations than *in situ* parathyroid adenomas, which may be related to a higher incidence of multiglandular disease, more severe hypercalcemia, and missed imaging studies (6, 7). Nonetheless, ectopic parathyroid glands remain a diagnostic and surgical challenge for patients with PHPT, contributing to initial surgical failures and persistent or recurrent PHPT (8). Surgery remains the preferred treatment for PHPT caused by EPA. Preoperative localization is crucial for ensuring the safety and efficacy of surgery, particularly in the era of minimally invasive procedures.

EPA can be found in various locations ranging from the skull base to the mediastinum. Ectopic inferior parathyroid glands are more common than superior glands due to their longer and more variable embryological origins (9). Superior ectopic parathyroid glands are frequently located near the tracheoesophageal groove or the posterior mediastinum (10). Ectopic inferior parathyroid glands are predominantly found in the mediastinum, accounting for approximately 38% of cases. However, they may also be confined within the thyroid gland itself in approximately 18% of cases (11). Moreover, ectopic parathyroid glands may occur in the vicinity of the vagus nerve, the foramen lacerum, the retropharyngeal space, or even within pulmonary tissues, leading to persistent PHPT (12–14).

To our best, only two cases of parathyroid adenomas located within the sternocleidomastoid muscle have been reported (15, 16). The two patients had a history of thyroidectomy due to goiter and medullary thyroid carcinoma respectively. Preoperative localization of lesion was performed by SPECT/CT imaging. And the localization of the lesion within the sternocleidomastoid muscle was confirmed by pathological examination. Two potential explanations have been proposed for this phenomenon. The first hypothesis suggests that parathyroid adenomas result from inadvertent seeding of parathyroid tissue during surgery. Research findings suggest that surgical relocation of the parathyroid gland into the sternocleidomastoid muscle may, albeit rarely, lead to EPA (15, 17). Therefore, we speculate that inadvertent damage to parathyroid tissue during thyroidectomy might result in its inadvertent implantation into the sternocleidomastoid muscle, potentially leading to the formation of EPA in this location. Wu et al. (8) reported a case of endoscopic parathyroidectomy performed via the axillary approach, which subsequently led to the emergence of an EPA within the ipsilateral pectoralis major muscle. Their case lends partial support to the theory implicating thyroidectomy in the genesis of EPA. The second hypothesis involves physiological stimuli inducing proliferation of embryologically pre-existing parathyroid tissue (13). In the present case, although the patient underwent partial resection of the right thyroid lobe due to nodular goiter, the ectopic parathyroid adenoma grew within the left sternocleidomastoid muscle. Consequently, the direct correlation between the ectopic parathyroid adenoma and the surgery performed eight years prior cannot be definitively established. Concerning the presence of parathyroid tissue within muscle, the topic of autotransplantation of parathyroid tissue is inevitable. Although muscle tissue is frequently utilized for autotransplantation of parathyroid tissue, the occurrence of adenomatous transformation in autotransplanted parathyroid tissue remains exceptionally uncommon.

Currently, several imaging modalities are also available to localize parathyroid adenomas preoperatively. While no method is perfect, most parathyroid adenomas are now localized preoperatively by a variety of imaging modalities, often using a combination of techniques to allow for successful resection with minimal harm to the patient (12). Presently, it is recommended that at least two diagnostic modalities be performed for patients who have developed persistent PHPT, achieving success rates as high as 95%. However, there is no consensus on the optimal combination of diagnostic modalities for these cases (18). Tc-99m-MIBI scintigraphy, ultrasound, and CT are the most commonly used methods for evaluating EPA. Tc-99m-MIBI scintigraphy is a highly mature imaging modality for the localization and diagnosis of parathyroid adenomas, particularly effective for adenomas smaller than 10 mm in diameter. In unexplored patients, Tc-99m-MIBI scintigraphy demonstrates a sensitivity of 70% to 89% and a specificity of 88% to 100% for localizing EPA (9). When identifying adenomas in patients undergoing repeat surgery, Tc-99m-MIBI scintigraphy exhibits a sensitivity of 65% to 67% and a specificity of 100% (9). Overall, Tc-99m-MIBI scintigraphy outperforms ultrasound and contrast-enhanced CT scanning (19). Ultrasound examination is cost-effective, widely available, and radiation-free, making it particularly suitable for assessing EPA. Generally, the sensitivity of ultrasound examination depends on the size and location of the enlarged gland. Ultrasound is accurate in identifying parathyroid adenomas adjacent to the inferior thyroid pole and the posterior aspect of the thyroid gland. Reported sensitivities of ultrasound for detecting EPA range from 11% to 59% in previously unexplored PHPT patients, with a specificity of up to 100% (20). The main reason for low ultrasound sensitivity is that many ectopic locations cannot be detected by routine ultrasound examination, especially in areas such as the thymus, the tracheoesophageal groove, etc. Additionally, the misidentification of thyroid nodules and lymph nodes as parathyroid lesions on ultrasound may also affect the specificity of ultrasound examination (21). Parathyroid adenomas appear as discrete soft tissue masses near or adjacent to the thyroid gland on CT scans. The sensitivity of CT scanning for parathyroid disease mainly depends on the size of the enlarged gland rather than its location. Therefore, CT scanning is better suited to evaluate the posterior esophageal, tracheal, and mediastinal regions compared to ultrasound examination (22). Since CT has lower diagnostic accuracy for smaller lesions and cases with multiple culprits, it is more commonly used when Tc-99m-MIBI scintigraphy results are negative (23). Finally, in cases where conventional imaging yields negative results, functional imaging modalities like 18F-choline PET/CT and 11C-choline PET/CT can aid in the detection of EPA. Their notable advantage lies in their heightened sensitivity and specificity, particularly in detecting minute tumors and precisely pinpointing their locations within the body (24, 25). Regrettably, our patient did not undergo preoperative PET/CT imaging, thus preventing us from obtaining PET/CT images of the EPA situated ectopically on the sternocleidomastoid muscle.

## 4 Conclusion

The diagnosis of PHPT caused by EPA remains challenging. Multimodal imaging examinations aid in the localization and diagnosis of EPA. In this case, we report a very rare instance of an EPA arising within the sternocleidomastoid muscle, and the unusual presentation on early Tc-99m-MIBI scintigraphy adds an intriguing aspect to the case.

## Data availability statement

The original contributions presented in the study are included in the article/supplementary material. Further inquiries can be directed to the corresponding author.

## Ethics statement

The studies involving humans were approved by Guangzhou First People’s Hospital Ethics Committee. The studies were conducted in accordance with the local legislation and institutional requirements. The participants provided their written informed consent to participate in this study. Written informed consent was obtained from the individual(s) for the publication of any potentially identifiable images or data included in this article.

## Author contributions

HS: Writing – original draft. ZJ: Writing – original draft. JX: Writing – original draft. JL: Writing – original draft. XZ: Writing – review & editing.
